# Bio-efficacy, physical integrity, use and attrition of long-lasting insecticidal nets under operational conditions for malaria prevention in Ghana

**DOI:** 10.1371/journal.pone.0275825

**Published:** 2022-10-14

**Authors:** Stephen Kantum Adageba, Edmund Wedam Kanmiki, Victor Asoala, Abraham Rexford Oduro, Philip Kweku Baidoo

**Affiliations:** 1 Navrongo Health Research Centre, Narvongo, Upper East Region, Ghana; 2 Department of Theoretical and Applied Biology, Kwame Nkrumah University of Science and Technology, Kumasi, Ghana; 3 Institute for Social Science Research, The University of Queensland, Indooroopilly, Queensland, Australia; 4 Poche Centre for Indigenous Health, The University of Queensland, Brisbane, Australia; 5 Research and Development Division, Ghana Health Service, Accra, Ghana; Fundação Oswaldo Cruz Centro de Pesquisas René Rachou: Fundacao Oswaldo Cruz Instituto Rene Rachou, BRAZIL

## Abstract

**Background:**

Malaria remains a public health challenge in endemic countries of the world. The use of Long-lasting Insecticidal Nets (LLINs) is one of the major ways of malaria vector control. Recent evidence however suggests some LLINs are unable to maintain their effectiveness over their useful life span. This study assessed the bio-efficacy, physical integrity, use and attrition at 6 and 12-months post-distribution of LLINs (LifeNet).

**Methods:**

Following a mass distribution of LLINs in the West Mamprusi District of the North-East region of Ghana in 2018, a total of 147 LLINs were sampled for physical integrity and attrition assessment using hole size and the number of holes as a measure of the proportionate hole index (pHI). Bioassays were conducted on sixty randomly selected LLINs using the WHO guidelines for bio-efficacy testing (cone tests), (20 each at baseline, midline and endline) over a one-year study period. Bed net ownership and use as well as malaria vector resistance status were also assessed.

**Results:**

Findings indicate high bio-efficacy of approximately 100% average mortalities of mosquitoes at baseline, 6-months and 12-months post-distribution. A small proportion of LLINs (0.8% and 5.6% at the 6 and 12-months surveys respectively) were damaged beyond maintenance while 62.4% and 62.7% of LLINs were used the night before the survey for 6 and 12-months post-distribution respectively. Households with electricity were less likely to use LLINs compared to those without electricity (P-value = 0.016, OR = 0.39). There were 20 fewer LLINs recovered at the 12-months relative to the 6-months resulting in 14.3% attrition rate. Susceptibility testing showed high pyrethroid and organochlorine resistance (18%, 67.5% and 3.8%) to local malaria vectors respectively), whereas organophosphates and carbamates recorded vector susceptibility of 100% for pirimiphos-methyl and 98.7% for bendiocarb.

**Conclusion:**

Biological efficacy, physical integrity and net attrition during the study period were in conformity with respect to the WHOPES one year net use. LLINs remained effective after one-year of usage. Net ownership was high in the study households. There should be continuous and regular distribution campaigns to maintain high coverage.

## Introduction

Malaria is a major health problem in Sub-Saharan Africa, and its prevention, treatment and control account for up to 40% of public health expenditure in many endemic countries [1]. The utilization of Insecticide-treated mosquito nets (ITNs) is an important tool for malaria prevention [2]. It is estimated that 68% of the 663 million cases of malaria prevented between 2000 and 2015 is through the increased use of bed nets [3]. In sub-Saharan Africa, an estimated US$ 900 million has been reported to be saved due to declining malaria, with US$ 610 million of those savings attributed to the use of bed nets [4]. The reduction in malaria mortality within the last fifteen years has also contributed to a 1.2 years increase in life expectancy [5].

There are however, reports of increasing insecticide resistance that is hampering this progress [6] with widespread malaria vectors being resistant to pyrethroids, the only category of insecticides presently accepted for use in bed nets [7]. A long-lasting insecticidal net (LLIN) is designed to remain potent for three or more years without the need for re-treatment with at least twenty washes [8]. However, evidence suggests that some LLINs are unable to maintain their effectiveness over their useful life span. Indeed, many cases of pyrethroids insecticide resistance have been documented [9]. This makes it vital to frequently re-examine the usefulness of LLINs to guide current vector control and resistance management policies [10]. The efficiency of LLINs hinges on the period of insecticidal action. Indications are that, several washes of LLINs make them lose their ability to kill mosquitoes in areas of high pyrethroid resistance [11]. The World Health Organization (WHO) as a matter of urgency called on the national malaria control programs in endemic countries to frequently evaluate the effectiveness of LLINs by assessing their attrition, durability, bio-efficacy and wholeness. Net attrition refers to the number of nets lost to follow up as a result of varied reasons. The WHO has supported the use of 11 diverse LLINs varieties with various types of pyrethroids for their treatment [12]. Polyester or polyethylene LLINs are recommended by the World Health Organization Pesticide Scheme (WHOPES). Polyester nets are typically smooth and soft to the touch, with high user acceptance, but they lack mechanical strength, with physical integrity rarely lasting more than 2–3 years. Polyethylene nets are more resistant than polyester nets in general. However, they frequently require heat treatments or additional time for insecticide regeneration, and they are typically rough to touch, both of which contribute to lower acceptability [13]. LifeNet is the target LLIN brand used in this study. This brand is the first of its kind to combine the power-driven strength of polypropylene and the efficacy of deltamethrin using the original incorporation procedure of 333/TC incorporated into 100 denier poly-filament polypropylene fibers at a dose of 8.5g AI/kg, corresponding to 340mg of deltamethrin per m2 LN [14]. The net has a mesh size of 21 complete holes per cm^2^ as a minimum, with an average of 21–29 holes per cm^2^ [14]. The minimum bursting strength of the fabric is declared as 450 kPa, which achieves biological activity beyond 35 standard washes with a multifilament construction to resist tear but remain soft to touch [15].

Ghana’s national malaria control program is using free distribution campaigns in targeting populations at risk of malaria, particularly in high malaria transmission communities. This leads to high rapid community-level coverage of bed nets across the country. However, little is known about the durability of these nets over time under operational conditions. This study assessed the bio-efficacy, physical integrity, use and attrition at 6-and 12-months post-distribution in the North East region of Ghana. The findings of this study will contribute to guiding malaria control efforts in Ghana and other countries where malaria is still endemic.

## Materials and methods

### Study setting

This study was conducted in the West Mamprusi district of the North-East region of Ghana. This district is one of the six districts in that region. The North-East region is located within the savanna ecological zone and like most parts of Ghana, has a high prevalence of malaria and intense transmission [16]. The West Mamprusi district was selected for this study because it is typical of most areas within the savanna ecological belt thus, research from this area has relevance for most areas within this ecological zone across sub-Saharan Africa. The study was coordinated by the Navrongo Health Research Centre [17], a center of excellence in health research where the researchers are based.

### Study design

This was a community-based prospective longitudinal study. Using the WHO guidelines, a household census was conducted in all the study communities before net distribution [18]. A standardized study questionnaire was used to ask about LLIN/ITN ownership and use in the study households. In this study, nets distributed freely by the National Malaria Control Program (NMCP) through mass campaigns were all LifeNets and were categorized as Long-Lasting Insecticidal Nets (LLINs) while some few Polyesters and Perma-Nets, found in the households were regarded broadly as Insecticide-Treated nets (ITNs). Net usage was also determined by asking net owners whether they slept under nets the night before the interview. If no member of the household slept under the net the night before the survey day, it was classified as non-used. The study team maintained comprehensive records of study participants that included information on the community, household head, adult population and children in the household, and the number of nets and sleeping places in the household. In all cases, codes were used to preserve participant anonymity and confidentiality.

### Sampling and sample size estimation

A two-stage cluster sampling using household census data from the mass distribution of LLINs in the study site was used. First stage was the random sampling of five communities within the district which was then followed by random sampling of households within those communities. The sample size for estimating net fabric integrity and attrition rate was estimated based on a conservative attrition rate to give room for any unforeseen participant loss to maintain enough participants left to monitor till the end of the study. Using the above criteria, a sample size of 147 LLINs was used for determining the attrition rate, fabric integrity and 20 nets for assessing the bio-efficacy [19].

There were two rounds of data collection during which the same LLINs were assessed at 6 -months and 12-months of use in the field. Six-month interval was deemed appropriate based on WHO recommendation for assessing the effective lifespan of nets durability [18]. Twenty nets were randomly selected and taken in each of the two cycles of data collection as well as the baseline for the bio-efficacy testing. These nets were often replaced by new nets of the same brand to ensure those study participants were not denied nets for their use. Nets replaced after the 6-months sampling for the bio-efficacy testing were not resampled during the 12-months survey. A sample size of 147 nets was randomly marked and chosen to be observed for the two sampling periods (June and December 2018) for data collection and to determine fabric integrity and net attrition rate.

### Insecticidal durability assessment

Bio-efficacy testing was done by cutting pieces from sampled nets from various points to determine whether the insecticide in the roof and sides of the net are causing mosquito mortality or not. Five net pieces, each 25 cm x 25 cm in size, were cut from five positions 1 to 5 of these nets specified by the WHO pesticide evaluation scheme [20]. Laboratory-reared female *Anopheles gambiae* s.s. Kisumu strain was used for the bioassay. Bioassays were done on all the five net pieces, placing one WHO cone on each netting sample. Five laboratory-bred, 3 and 4-day old, non-blood fed female *An*. *gambiae* s.s. Kisumu strain was introduced into each cone and exposed for 3 minutes. The test was carried out twice on each net piece. Fifty mosquitoes were exposed on each net resulting in a total of 3000 mosquitoes (3 time points–baseline, 6-months and 12-months, 20 nets at each time point, 5 positions per net, 5 mosquitoes per piece, 2 tests on each piece) being tested. After the exposure, mosquitoes were gently removed from the cones and kept separately in plastic cups. The analysis was based on the average mortality from all five cones and the 2 repeated tests per net. Mosquitoes exposed to untreated nets were used as a control. The bioassays were conducted at 25±2°C and 75±10% relative humidity (RH) at the Entomology Laboratory of the Navrongo Health Research Centre.

### Assessment of attrition and fabric integrity

As expected, at baseline, immediately after the free mass distribution of LLINs was carried out, net survivorship was 100% whilst net attrition was zero (0%). Study households were visited after 6-months post distribution and study nets were tracked. Confirmation of the coded nets were visually checked and if the study nets were not in the households, reasons for their losses were determined. At 6 and 12-months post distribution, nets physical integrity was evaluated through examination of the study nets with the codes on them. Damages in the study nets were determined by draping the nets over a frame and counting the number of holes and measuring their sizes [21]. This was recorded based on the four major holes categories smaller than a thumb (0.5–2 cm), larger than a thumb but smaller than a fist (2–10 cm), larger than a fist, but smaller than a head (10–25 cm) and larger than a head (> 25 cm) [14].

Considerations were also taken with regards to the five different hole locations on the net by dividing the side panels of the net into four zones from top to bottom, each measuring 37.5 cm, and counting holes in the roof separately as a fifth location on the net. Proportionate hole index (pHI) was calculated using the hole size classifications base on WHO guidelines [8].

### Insecticide susceptibility test

Susceptibility of malaria vectors was examined using the WHO Tube test procedure to determine insecticide resistance on *An*. *gambiae s*.*l* [8]. A total of 840 *An*. *gambiae s*.*l*, 3–5 day emerged from field collected larvae from the study site were used in the susceptibility testing at baseline of the study. One hundred twenty (120) lively female mosquitoes were suctioned (in clusters) from a mosquito confine into the six green-specked holding tubes through the filling gap in the slide, to give six recreate tests of 20 mosquitoes for each tube. The following insecticides at various concentrations were used in the test; Deltamethrin (0.05%), PBO+Deltamethrin (5.0–0.05%), Alphacypermethrin (0.5%), DDT (4.0%), Bendiocarb (0.1%), Malathion (5.0%) and Pirimiphos-Methyl (5.0%). This bioassay was used to quantify mosquito death rate to an established strength of these insecticides, either with a sharp concentration or with intensity concentrations. Average knock-down rates were determined after 60 minutes of exposure to the insecticide while average mortality was determined after a 24-hour holding period of the exposed mosquitoes as shown in Table 6.

### Data analysis

The questionnaire data were field-checked and validated; inconsistencies were corrected before analysis in STATA software. Basic descriptive statistics were used to describe the composition of variables. We specifically investigated the differences between cross-sectional surveys (6-months, and 12-months surveys) in the proportion of people reporting sleeping under a net last night, the presence of tracked nets (attrition rates), the bio-efficacy and physical integrity of nets at the successive cross-sectional surveys. To test whether these differences were statistically important, the mean comparison test (t-test) was applied to uncover whether net use or net attrition changed over time. Bio-efficacy remained high throughout, so we simply tested whether there was a statistical difference in the mosquito mortality at baseline versus 12-months using a “t-test.” Furthermore, multivariate logistic regression models were used to examine the predictors of LLINs utilisation and physical integrity of LLINs. Outcome variables were coded 0 and 1 for use and non-use or serviceable and non-serviceable respectively. Predictor variables involved in regression analysis are; educational attainment of net user, number of people in household, number of sleeping places in household, number of LLINs in household, availability of electricity in household, community and survey period. p-values, odds ratios and confidence interval have been reported. Statistical significance has been set at p-value< 0.05.

### Ethical statement

Ethical clearance for this study was obtained from both the Kwame Nkrumah University of Science and Technology Medical Research Ethics Committee and Navrongo Health Research Centre Institutional Review Board (NHRCIRB). Written Inform consent was obtained from all study participants before their involvement in the study.

## Results

### Demographic characteristics of households

Table 1 presents descriptive statistics of households involved in this study. A total of 147 households were surveyed in the five (5) study communities. Household heads, net users or representatives were interviewed on several aspects of ITN/LLINs. The average number of household members was 8.48 whilst the average number of sleeping places in a household was 3.76 (range; 1–11). All households surveyed owned a net; with the least being 2 nets and the highest being 15 nets per household respectively. The number of people that slept under a net the night proceeding the day of the survey were 62.4% and 62.7% for six and twelve-months post-distribution respectively.

**Table 1:** Descriptive statistics of the demographics of study households (n = 147).

Variable	Number	Sample mean	Standard deviation
Number of people in all selected households	1246	8.48	4.56
Number of adults in all households	639	4.35	2.86
Number of adolescents in all households	341	2.32	1.76
Number of children under-five in all households	266	1.81	1.67
Number of sleeping places in all households	553	3.76	1.89
Number of all types of bed nets owned by a11 households	792	5.39	2.92
Number of LLINs owned by all households	763	5.19	2.85

### Bioassay evaluation

The knockdown and mortality at baseline and midline were 100% whilst the knockdown rate at the endline (12 months) was 99.80% with a mortality of 100% at 60 minutes of exposure and 24 hours of holding time after exposure respectively for knockdown and mortality rates. The independent sample t-test showed no significant difference between the baseline and the endline (p-value = 0.178).

### Ownership and use of ITNs/LLINs

All the households involved in the study had at least one bed net. The 147 households surveyed owned a total of 792 bed nets (both ITNs and LLINs) with a mean of 5.4 (± 2.9) nets per household. An analysis of people per net in households showed a mean ratio increase from 1.83 during 6-months to 2.09 at 12-months. Indicating a statistically significant increase in the number of people to a bed net (p-value = 0.028). Table 2 provides more details on the people per net ratio in study households.

**Table 2:** Ratio of people per net in households (t-test).

Period	No. Households	Mean ratio	Std. Err.	Std. Dev.	[95% Conf. Interval]	
6 months	147	1.83	0.08	0.99	1.66	1.99
12 months	145	2.09	0.08	1.02	1.92	2.25
Combined	292	1.96	0.06	1.01	1.84	2.07
Difference		0.26	0.12		0.03	0.49

Pr (|T| > |t|) = 0.0281.

Overall, LLINs use among study participants during the first six months post-distribution survey was 62.4%. Post twelve months survey had net use among study participants to be 62.7%. Eighty-three individuals (62.4%) residing in the surveyed households slept under LLIN the night before the survey at six months while 79 (62.7%) slept under the study net the night before the survey at twelve months post-distribution. Table 3 shows the multivariate regression analysis of factors associated with LLIN use the night prior to the survey. Results show that those belonging to housholds with electricity were less likely (OR = 0.39, p-value = 0.016) to use LLIN compared to those without electricity. All other factors including educational attainment, number of people in a household, number of sleeping places, number of LLINs, community and the survey period were not significantly associated with LLINs use.

**Table 3:** Logistic regression of factors associated with LLIN utilization.

VARIABLES	Odds Ratio	95% Confidence Interval
**Educational Attainment (Compared with None)**		
Primary/Junior High School	1.27	(0.58–2.78)
Secondary School and Above	0.83	(0.34–2.06)
**Number of People in Household (Compared with less than 4)**		
4–6	1.16	(0.44–3.12)
7–9	2.44	(0.80–7.48)
10 and above	1.51	(0.44–5.14)
**Number of Sleeping Places in Household (Compared with less than 3)**		
3–5	1.63	(0.82–3.24)
6 and above	0.56	(0.19–1.70)
**Number of LLINs in Household (Compared with less than 3)**		
3–5	0.81	(0.39–1.67)
6 and above	0.89	(0.32–2.49)
**Electricity in the Household (Compared with No Electricity)**		
Has Electricity	0.39*	(0.17–0.88)
**Community (Compared with Duu)**		
Janga community	0.87	(0.28–2.67)
Kpesenkpe community	0.83	(0.26–2.66)
Mishio community	1.27	(0.39–4.18)
Nasia community	0.64	(0.17–2.35)
**Survey Period (Compared with 6 months)**		
12 Months	1.07	(0.60–1.91)
Constant	2.44	(0.50–11.82)
Observations	256	

*** p<0.001, ** p<0.01, * p<0.05.

**Fig 1:**
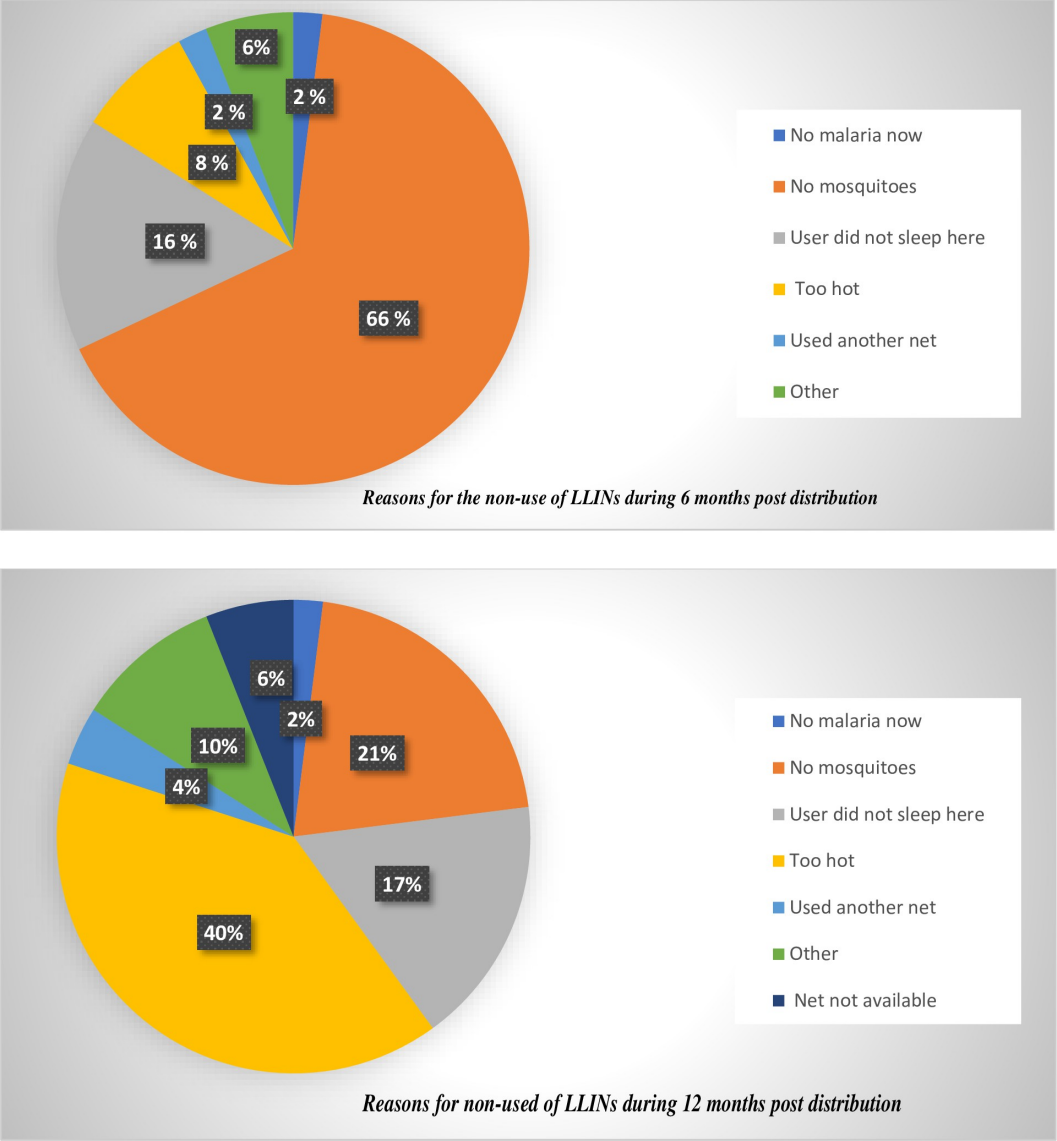
Reasons for not using LLINs.

The study participants gave several reasons why they do not sleep under the study LLINs every night (see Fig 1). According to 66% of those who did not use the LLIN last night, there were no mosquitoes while 16% indicated that they slept somewhere else during the night prior to data collection. Some 18% indicated other factors accounting for their non-use of LLINs during the first six months after net distribution as shown in Fig 1. During the 12 months post distribution, the weather being too warm accounted for 40% of non-use of the study nets, a further 21% of the participants indicated the absence of mosquitoes while 17% of the participants slept in a different place other than the study household.

### Physical integrity of LLINs

The LLINs which developed holes of various categories out of the 133 nets that were examined are shown in Table 4. Overall, net holes for the first six months post-distribution were 48 (36.1%) and 99 (78.6%) 12 months post-distribution respectively. The category of holes between 0.5 and 2.0 cm were mostly found around the lower portions of the LLINs and these were the majority.

**Table 4:** Physical integrity of LLINs with holes of different size categories.

Hole size category		Duration (Months)			
		6 months post-distribution of nets		12 months post-distribution of nets	
Hole size category	Hole size (cm)	Nets with holes (%)		Nets with holes (%)	
1	0.5 ‒ 2.0	22 (16.4)		45 (35.7)	
2	2–10	19 (14.2)		39 (31.0)	
3	10–25	5 (3.7)		12 (9.5)	
4	> 25	2 (1.5)		3 (2.4)	
Total		**48 (35.8)**		**99 (78.6)**	

The LLINs at the 6 and 12-months after distribution had various pHIs. The number of LLINs in the “excessively torn” class increased depending on its age and frequency of use. ‘Good’ and ‘damaged’ classes of nets were considered as “serviceable” while the “too torn” nets are classified as “unserviceable”. Based on the field data, 1 net and 7 nets fell in the unserviceable categories in 6 and 12-months post-distribution respectively. Table 5 is the regression results of correlates of having a pHI within the serviceable category. LLINs in households with higher number of people were less likely to be serviceable (p-value<0.05). All other variables were not significantly associated with having a serviceable LLINs or not.

**Table 5:** Logistic regression of factors associated with pHI of LLINs.

Variables	Odds Ratio	95% Confidence Interval
**Educational Attainment (Compared with None)**		
Primary/Junior High School	0.92	(0.08–10.20)
Secondary School and Above	-	
**No. of People in Household**	0.68*	(0.51–0.92)
**No. of Sleeping Places in Household**	2.75	(0.94–8.03)
**No. of LLINs in Household**	1.36	(0.68–2.70)
**Electricity in the Household (Compared with No Electricity)**		
Has Electricity	1.33	(0.15–12.14)
**Community (Compared with Duu)**		
Janga	0.35	(0.02–6.29)
Kpesenkpe	0.86	(0.03–25.31)
Mishio	2.29	(0.05–114.79)
Nasia	-	
**Survey Period (Compared with 6 Months)**		
12 Months	0.25	(0.02–3.20)
**Constant**	45.58	(0.72–2,874.60)
Observations	217	

*** p<0.001, ** p<0.01, * p<0.5.

NB: Number people in household, number of sleeping places and number of LLINs are treated as continuous variables.

### Insecticide susceptibility tests of *An*. *gambiae* using WHO tube test

Results of the susceptibility test for 840 *An*. *gambiae* s.l is summarized in Table 6. The test results of all seven insecticides and control mortality were less than 5%, which did not require Abbott’s formula to correct the experiment. Organophosphates and Carbamates showed efficacy, with Pyrethroids, Organochlorines and synergist recording higher levels of resistance. Field *An*. *gambiae* s.l collected from the study district was susceptible to Pirimiphos-methyl and Bendiocarb insecticides with mortalities of 100% and 98.7% respectively. However, mosquitoes from all sampled sites of the study area showed resistance to Deltamethrin, PBO + Deltamethrin, Alphacypermethrin and DDT. Malathion insecticide recorded 97.5% 24-hour mortality thus indicating a suspected resistance status. The WHO criteria were used to assess the level of resistance in the study communities [22], (98–100%) mortality indicates susceptibility, (80–97%) mortality suggests suspected resistance that needs to be confirmed and (< 80%) mortality suggests resistance.

**Table 6:** Insecticide susceptibility tests status of wild *An*. *gambiae*.

Insecticide	Concentration (%)	Knockdown (60 mins)	Control knockdown	Final test mortality (24 hrs)	Number of mosquitoes tested	Control mortality	Susceptibility status
Deltamethrin	0.05	36.4	0.0	18.2	120	0.0	Resistant
PBO+Deltamethrin	5.0 + 0.05	92.5	0.0	86.3	120	0.0	Resistant
Alphacypermethrin	0.5	92.5	0.0	67.5	120	0.0	Resistant
DDT	4.0	5.0	0.0	3.8	120	0.0	Resistant
Bendiocarb	0.1	98.7	0.0	98.7	120	0.0	Susceptible
Malathion	5.0	81.0	0.0	97.5	120	0.0	Suspected Resistant
Pirimiphos-Methyl	5.0	55.0	0.0	100.0	120	0.0	Susceptible

### LLINs attrition

Net attrition in the context of this study refers to the loss or unavailability of LLINs from the study households due to wear and tear or other factors. During six months post-distribution of LLINs, every study household received a follow-up visit and visual verification of all study LLINs that were distributed to them was done. Study nets that were no longer in the households for use were either discarded because they were badly damaged (1%) and can no longer be used or were gifted to others (13.61%) to use. The overall attrition rate from the start of the study to the end of the study was 14.3% (21). With almost all (20 nets) due to loss, gifting out to relatives or theft.

## Discussion

This study examined the biological efficacy, physical integrity, household ownership and use of LLINs, and determined the insecticide resistance status of malaria vectors and attrition of LLINs under operational conditions. Household ownership of LLINs was 100%, however, utilization was found to be 62.4% and 62.7% for 6 and 12-months post-distribution of LLINs. This high ownership of LLINs in households is obviously due to the free mass distribution of LLINs immediately before the study commenced. In the Eastern Region of Ghana, 91.3% ownership was found after thirty-one months post-distribution of LLINs [23]. Based on individual household ownership levels, it is important for the National Malaria Control Programme of Ghana to begin plans for LLINs distributions after a one-year post distribution of free mass campaigns as bed net ownership begins to effectively change after 12 months post-distribution.

Results from this study demonstrate that bed net use, for the two periods of data collection was similar to a previous study in the Upper East region of Ghana where bed net use the night prior to the survey was 62% [24]. The fact that households with access to electricity were found to be less likely to use bed nets suggest that availability of electricity provides avenues for using other preventive strategies to avoid mosquito bites including electric fans and other repellents.

Results from this study demonstrate that efforts by the Ghana Health Service and the National Malaria Control Programme and other Non- Governmental Organizations (NGOs) in improving the ownership and use of bed nets in Ghana are achieving the desired target coverage of 80% or more, but there are critical disparities between the possession and use of LLINs. This was obvious in this current study where household ownership of LLINs was high, but usage was moderate. The main reasons behind not utilizing a mosquito net, even with access to one were due to discomfort from heat, the perception of absence of malaria vectors and the net user sleeping elsewhere other than his or her home. Minor reasons, among others, were due to no malaria perception, net not available and use of other nets. These findings are consistent with what has been reported elsewhere [25].

Overall, inconvenience and heat were the predominant reasons behind non-use in most cases and discomfort as well as mosquito densities. Findings from this study showed that the predominant reason for non-use of LLINs the night preceding the survey was due to low mosquito vector densities. During the 12-month post-distribution survey, 41% of study households failed to use their LLINs due to discomfort of the hot weather (heat) out of the ten reasons for the non-use of the study LLINs.

Assessing the biological efficacy of the study nets at 6 and 12-months post-distribution showed good efficacy and thus recorded high mortality in cone bioassays. Results of bioassay at baseline and midline of this study recorded 100% mortality, with the endline recording a mortality rate of 99.80%, thus indicating high bioassays results. All the LifeNets withdrawn from the study communities for the bioassays met the WHO cutoff value of ≥ 80% mortality. A study in Tanzania examined the biological efficacy and physical integrity of 3 different LLIN brands: Olyset, permethrin incorporated in 150 denier polyethylene; PermaNet 2.0, deltamethrin coated on 100 denier polyester; and NetProtect, deltamethrin incorporated in 110 denier polyethylene [15]. Their findings revealed that the lifespan of pyrethroid-treated LLINs varies dramatically. The LLINs passing WHO bioefficacy standards had been above 80% for NetProtect and PermaNet 2.0 and below 80% for Olyset. NetProtect and PermaNet had comparable physical integrity after three years of use, with a better percentage of serviceable nets relative to Olyset. The LLINs in their study had a median functional life of less than three years [15].

There was no significant difference in mortality irrespective of the part of LLIN used in the test. Understanding net loss (attrition), biological efficacy of LLINs distributed free of charge by National Malaria Control Programme should be regularly monitored in malaria transmission regions to advise the LLINs procurement and replacement [26].

In this study, most nets were still in good physical condition after one year. A study from the South-East region of Madagascar however found a high loss of physical integrity after two years post distribution[27]. In this study, out of 133 LLINs examined for physical integrity in the six months post-distribution, only one (0.8%) was physically damaged and could not be used for the protection against malaria vector control compared to some other LLINs that developed holes more quickly. Yorkool® LLIN, Olyset® Net, and Interceptor® are reported to develop holes more quickly than other brands [28–30]. In the case of Life Net®, studies found that, it had good physical integrity but had higher rates of being given away to others, which could indicate that user preferences or acceptability differed amongst the many LLIN brands distributed [29]. One-year post-distribution recorded seven (5.6%) LLINs that were no longer in good condition and were therefore discarded. Findings of this study are relatively low compared to similar results from Zambia, where 9.6% of nets after 12-months post-distribution were ‘too torn’ [31]. Bed nets get damaged through holes development over time. A similar study in Western Uganda showed a large number of net holes with increased period of use [32]. In the current study, 99.2% and 94.4% of LLINs were in good condition for use against malaria vector control after 6 and 12-months post-distribution, contrary to a study in Ethiopia that had some LLINs users complained of their nets been old and could no longer protect them against mosquitoes bits at 12- months post-distribution [33]. The proportion of LLINs with holes in them during 6-months post-distribution was 35.8% (48/133) and 78.6% (99/126) which is similar to a study in Uganda where one-year post-distribution resulted in 45–78% of nets being physically damaged [34]. Most of the net holes were situated on the lower parts of the nets which are often the part tucked under the sleeping mat or mattress. Household conditions like the type of sleeping place, materials used in building houses and personal care for the nets are known impact on the physical integrity of nets over time [35]. Classification of LLINs by the WHO into “Good”, “Damaged” and “Too torn” [36] using pHI values of all nets with holes showed an estimated 93.6% of nets in the first two categories, “Good” and “Damaged” considered serviceable and 6.4% of these nets were considered “Too torn” and thus discarded due to their physical damage beyond repairs.

Entomological monitoring and evaluation of malaria vectors for susceptibility status to insecticide resistance are recommended by WHO in all countries that use LLINs or indoor spraying as major interventions for malaria control [22]. The degree of worldwide insecticide resistance reportage has improved over time. Despite this, there are some malaria-endemic regions for which there is no information for guiding interventions against vector control.

Pyrethroids remain the key insecticide class for use against malaria control as they are the main class prescribed for use on LLINs [37]. Some countries in Africa that have operationalized insecticide resistance monitoring for 9 years have identified pyrethroid resistance in certain regions [38]. Several countries in sub-Saharan Africa have identified insecticide resistance from different insecticide classes. Since few investigations have attempted to measure the insecticide resistance quality in field vector populations, it is hard to know whether this extraordinary resistance phenotype is uncommon or symptomatic of the status of pyrethroid insecticide resistance in malaria vectors in Africa [39].

In an ideal situation, the finding of pesticide resistance would prompt a change in insecticide class as a key component of a proactive management strategy to combat resistance. Because only pyrethroids are presently recommended for treating bed nets and use as an alternative insecticide class for indoor residual spraying (IRS), this frequently brings about higher program costs [7]. Along these lines, information acquired from routine resistance assessment should provide adequate evidence for insecticide-based vector control procedures.

The level of insecticide resistance from the findings of this study presents an alarming challenge to the success of interventions geared towards malaria control. Since few studies have tried to measure resistance quality in field vector populations in Ghana, it is unclear whether this striking insecticide resistance phenotype is unresolved or symptomatic of the status of pyrethroid resistance malaria vectors in Africa as a whole. Essentially, in Ghana, pyrethroid resistance sets off a change to the utilization of the organophosphate insecticide Actellic (primiphos methyl) for IRS that was related with a recognizable effect on key pointers of malaria transmission.

In this study, monitoring of insecticide resistance status of local populations of malaria vectors was conducted with four classes of insecticides: Organochlorines, Pyrethroids, Organophosphates and Carbamates. The findings of this study indicated vector resistance to pyrethroids, organochlorines and a synergist of deltamethrin, thus failing to meet the WHO mortality of 98–100% after an hour of exposure to WHO impregnated test papers and holding exposed mosquitoes for a 24-hour period. The rapid growth of deltamethrin resistance in An. gambiae *s*.*l* populations was demonstrated in this study. These findings are critical for developing vector surveillance and control methods to combat insecticide resistance. In these locations, trials of novel vector control techniques, such as new generations of LLINs that use alternative classes of insecticide or synergists, are needed to determine the epidemiological and entomological impact in comparison to pyrethroid LLINs. Organophosphate (pirimiphos-methyl) and carbamate (bendiocarb) recorded 100% and 98.7% mortalities respectively. However, mosquitoes from all sampled sites showed resistance to Deltamethrin, PBO + Deltamethrin, Alphacypermethrin and DDT. Malathion insecticide recorded 97.5% 24-hour mortality thus indicating a suspected resistance status. These findings support a recent study in Ghana in which bendiocarb showed low resistance intensity to malaria vectors with a 98.7% average mortality after 24 hours holding period [40]. The results of this study also confirms previous studies conducted in Ghana on insecticide resistance of malaria vectors [41].

Long-lasting insecticidal net attrition gives vital insights in terms of the survival, utilization and quality of the nets with respect to the physical integrity of the net fabric. Results of this study showed the main cause of net attrition was due to the removal of study LLINs from the households as in a similar study in sub-Saharan Africa [42]. Other studies have found that most of the LLINs were either torn or were not in the households for use within the stipulated 3 years as a result of low net durability, thus suggesting that the serviceable life of LLIN was really closer to two, instead of 3 years [34,35].

## Conclusion

The results of the study highlight good insecticide efficacy of LifeNet © at baseline, 6-months and 12-month post-distribution and thus met WHOPES requirements. The high possession and utilization of LLINs in the West Mamprusi District may be due to different interventions that have been executed in the area by the Ghana Health Service (GHS) and the National Malaria Control Programme. Net attrition was found to be mainly due to nets being given out to others or lost and no longer available for sleeping under. Susceptibility of local malaria vectors to classes of insecticides showed high resistance to pyrethroids, which is the main insecticide recommended by the WHO for impregnation of LLINs. Custom-made efforts and instructive crusades are required to guarantee the steady utilization of LLINs.

## Supporting information

S1 File Study questionnaire. (PDF)

S1 Dataset (DTA)
