# Supplementary material for: Bio-efficacy, physical integrity, use and attrition of long-lasting insecticidal nets under operational conditions for malaria prevention in Ghana
Source: PLoS One. 2022 Oct 14;17(10):e0275825. doi: 10.1371/journal.pone.0275825 (PMC9565380; doi:10.1371/journal.pone.0275825)
Supplement: S1 File — (PDF) [file pone.0275825.s001.pdf]

MONITORING THE DURABILITY OF LONG-LASTING INSECTICIDAL NETS (LifeNet) UNDER OPERATIONAL CONDITIONS FOR MALARIA CONTROL IN THE WEST MAMPRUSI DISTRICT OF THE NORTHERN REGION OF GHANA

| **Section 1: I would like to ask you (head of household or adult > 18 years some questions about your household.** | | | |
|---|---|---|---|
| Q# | Questions and filters | (Circle response) | variable |
| 1.1 | Who is responding to the question? | 1. Head of household<br>2. User of net<br>3. Parent or guardian of user(s) of net<br>4. Other adult in household | RESPON |
| 1.2 | What is the highest level of education of the head of the household? | 1. None<br>2. Primary school<br>3. Secondary school<br>4. Higher<br>5. Others | EDUCA |
| 1.3 | Does your household have electricity? | 1. Yes<br>2. No | ELECT |
| 1.4 | What is the principal type of toilet facility used by members of the household? | 1. Own flush toilet<br>2. Shared flush toilet<br>3. Own pit latrine<br>4. Shared pit latrine<br>5. Bush or field<br>6. Others | TOILET |
| 1.5 | What is the principal household source of drinking water? | 1. Piped water into home<br>2. Protected well in home<br>3. Unprotected well in yard<br>4. Open well in yard<br>5. Protected well in yard<br>6. Unprotected public well<br>7. Protected public well<br>8. Tap in yard<br>9. Tanker truck<br>10. Boiled water<br>11. Public tap<br>12. Borehole<br>13. Surface water<br>14. Others | DWATER |
| 1.6i-iii | How many people slept in your household last night? (please enter number into appropriate box) | [ I ] | |

| | | | |
|---|---|---|---|
| 1.6i | Adults > 15 years | [ ] [ ] | NADULT |
| 1.6ii | 5- 15 years | [ ] [ ] | NADOLE |
| 1.6iii | < 5 years | [ ] [ ] | NCHILD |
| 1.7 | How many sleeping places were used last night in your household? ( including sleeping places outside and temporary spaces) | [ ] [ ] | PLACES |
| 1.8 | How many mosquito nets that can be used for sleeping does your household have? ( *probe for an nets currently not in use: stored, saved, still in packaging)* | [ ] [ ] | NUMNETS |
| 1.9 | Of the total number of mosquito nets, how many are LLINs (O*bserve*) | [ ] [ ] | NLLIN |
| **Section 2: LN status (for selected net identification \|__\|__\|__\|__\|__\| )** | | | |
| 2.1 | Is this net still in the household and can be used for sleeping under in the the household?<br>(Look for the net in the household, including those still in packaging or being used for another purpose) | 1. Yes ⟶ skep to section 3<br>2. No | LLNAV |
| 2.2 | If no, why is the net no longer available for sleeping under in the household? | 1. Net was damaged and thrown away<br>2. Net was given away to others<br>3. Net was stolen<br>4. Net was sold<br>5. Net is being used in another location<br>6. Net is being used for another purpose<br>7. Others, specify | NOLLIN |
| 2.3 | How many months ago did this net become unavailabe for sleeping under in the household? | 1. 0-6 months ⟶ End questionaire<br>2. > 6 months ⟶ End questionaire<br>3. Don't know ⟶ End questionaire | MONHS |
| Section 3: LNuse and handling ( for selected net identification \|__\|__\|__\|__\|__\| ) | | | |
| 3.1 | Has the net ever been used for sleeping under? | 1. Yes<br>2. No ⟶ End questionaire | EVERU |
| 3.2 | Was this net used last night to sleep under? | 1. Yes Skip to 3.4<br>2. No | LLNITE |
| 3.3 | If no, why did you not use the net last night? | 1. Too hot<br>2. Don't like the smell | |

| | | | |
|---|---|---|---|
| | | 3. Feel "closed in"<br>4. No malaria now<br>5. No mosquitoes<br>6. The net is too torn or old<br>7. Net not available<br>8. Used another net<br>9. User did not sleep here<br>10. Don't know<br>11. Others, specify | WYNOT |
| 3.4 | In the past week, how often was the net used? | 1. Every night (7 nights)<br>2. Most nights (5-6 nights)<br>3. Some nights (1-4 nights)<br>4. Not used at all (0 nights)<br>5. Don't know | UFREQ |
| 3.5 | How many adults (>15 years) slept under this net last night | \|___\|___\| | SADULT |
| 3.6 | How many children 5-15 years slept under this net last night? | \|___\|___\| | SADOLE |
| 3.7 | How many children < 5 years slept under this night last night? | \|___\|___\| | SCHILD |
| 3.8 | During which periods of the year is this net used to sleep under? | 1. All year<br>2. Only the rainy season<br>3. Only the dry season<br>4. Don't know | SEASON |
| 3.9 | Is this net ever used for sleeping under away from the main house? If yes, where? | 1. Taken to the fields<br>2. Taken to the beach<br>3. Taken to the forest<br>4. Taken to the farm hut<br>5. Other, specify<br>6. Not used away → Skip to Q# 3.11<br>7. Don't know → Skip to Q# 3.11 | UAWAY |
| 3.10 | During which periods of the year is this net used to sleep under away from the main house? | 1. All year<br>2. Only the rainy season<br>3. Only the dry season<br>4. Don't know | AWAYSE |
| 3.11 | Has this net ever been used over the following types of | 1. Cut wood | |

| | | | |
|---|---|---|---|
| | sleeping places? | 2. Foam mattress<br>3. Wooden bed frame (finished)<br>4. Wooden bed frame (sticks)<br>5. Metal bed frame<br>6. Bare floor or ground<br>7. Others, (specify)………………………… | SPLACE |
| 3.12 | Do you tuck the net at night? | 1. Yes<br>2. No<br>3. Don't know | TUCKIN |
| 3.13 | Has the net ever been washed? | 1. Yes<br>2. No ⟶ Skip to Q# 4.1<br>3. Don't know ⟶ Q# 4.1 | EWASH |
| 3.14 | When was the last time you washed your net? | 1. 1 week ago<br>2. 1 week to 1 month<br>3. 1-3 months ago<br>4. 3-6 months ago<br>5. > 6 months ago<br>6. Don't know | LWASH |
| 3.15 | What type of soap was used? | 1. None<br>2. Local bar soap<br>3. Detergent powder<br>4. Mix ( bar soap and detergent powder)<br>5. Bleach<br>6. Don't know | SOAPU |
| 3.16 | How long did the net soaked? | 1. Did not soak the net<br>2. < 1 hour<br>3. > 1 hour<br>4. Don't know | LTYME |
| 3.17 | Was the net scrubbed hard or beaten on a hard surface ( e.g. rocks, with sticks) | 1. Yes<br>2. No<br>3. Don't know | HWASH |
| 3.18 | Where was the net dried? | 1. Outside in the sun<br>2. Outside in the shade<br>3. Inside<br>4. Don't know | NDRIED |

| **Section 4: LN condition ( for selected net identification \| \| \| \| \|)** | | | |
|---|---|---|---|
| 4.1 | In the past month, have any new holes appeare in the net that you are aware of? | 1. Yes<br>2. No<br>3. Don't know | NHOLES |
| 4.2 | What caused these holes? | 1. Tore or split when caught on an object<br>2. Was burnt<br>3. Was caused by animals<br>4. Was caused by children<br>5. Don't know<br>6. In another way specify | CAUSES |
| 4.3 | How is the net found? (Observe) | 1. Hanging loose over sleeping place<br>2. Hanging tied in knot<br>3. Hanging folded<br>4. Visible but not hung up<br>5. Stored away | OBSERV |
| 4.4 | What type of sleeping place is the net hanging over? | 1. Reed mat<br>2. Cut wood<br>3. Grass<br>4. Foam mattress<br>5. Woodwn bed frame (finished)<br>6. Wooden bed frame (sticks)<br>7. Metal bed frame<br>8. Nothing<br>9. Others, specify | TPLACE |
| 4.5 | Where is it found? (Observe) | 1. Inside<br>2. Outside → Skip to Q# 4.9 | PLACE |
| 4.6 | What is the principal tye of flooring in the room where the net is found? | 1. Soil or sand<br>2. Tiles<br>3. Cement (including vinyl)<br>4. Cement<br>5. Carpet<br>6. Others, specify………………………… | FLOOR |
| 4.7 | What are the walls of the room in which the net is found made of? (Observe) | 1. Mud brick<br>2. Mud with wooden frame<br>3. Congrete | |

| | | | |
|---|---|---|---|
| | | 4. Wood<br>5. Straw<br>6. Corrugated<br>7. Lime-plastered<br>8. No walls (used outside)<br>9. Others | WALLW |
| 4.8 | What is the roof orceiling of the room in which the net is found made of ? (Observe) | 1. Grass thatch<br>2. Corrugated iron<br>3. Concrete<br>4. Reed mats<br>5. Wood<br>6. Tiles<br>7. Others, specify | ROOFM |
| 4.9 | Do you use an open flame for cooking heating or lighting where the net is found? | 1. Wood fire<br>2. Charcoal fire<br>3. Wax candle<br>4. Oil lamp with a glass<br>5. Oil lamp without a glass<br>6. Others, specify | COOKT |
| 4.10 | What types of holes are observed? | 1. Horizontal tears at bottom<br>2. Holes at hanging points<br>3. Open seams<br>4. Burnt holes<br>5. Holes from rodents<br>6. Whole section missing | TYPEHO |
| 4.11 | Number of holes of size 1 | Less than size of thumb (0.5-2cm) | |
| 4.11a | Number found in the roof | \| \| \| | 1.SIZER |
| 4.11b | Number found in the upper | \| \| \| | 1.SIZEU |
| 4.11c | Number found in the lower | \| \| \| | 1.SIZEL |

| | | | |
|---|---|---|---|
| 4.11d | Number found in the seams | \| \| \| | 1.SIZES |
| | | | |
| 4.12 | Number of holes size 2 | Larger than a thumb, smaller than fist (2-10 cm) | |
| 4.12a | Number found in the roof | \| \| \| | 2.SIZER |
| 4.12b | Number found in the upper | \| \| \| | 2.SIZEU |
| 4.12c | Number found in the lower | \| \| \| | 2.SIZEL |
| 4.12d | Number found in the seams | \| \| \| | 2.SIZES |
| | | | |
| 4.13 | Number of holes of size 3 | Larger than fist, smaller than head (10-25cm) | |
| 4.13a | Number found in the roof | \| \| \| | 3.SIZER |
| 4.13b | Number found in the upper | \| \| \| | 3.SIZEU |
| 4.13c | Number found in the lower | \| \| \| | 3.SIZEL |
| 4.13d | Number found in the seams | \| \| \| | 3.SIZES |
| | | | |
| 4.14 | Number of holes of size 4 | Larger than head (25cm-) | |
| 4.14a | Number found in the roof | \| \| \| | 4.SIZER |
| 4.14b | Number found in the upper | \| \| \| | 4.SIZEU |
| 4.14c | Number found in the lower | \| \| \| | 4.SIZEL |
| 4.14d | Number found in the seams | \| \| \| | 4.SIZES |
| | | | |

| | | | |
|---|---|---|---|
| 4.15 | Number of holes repaired | | |
| 4.15a | Number of stiched | \| \| \| | NSTITCH |
| 4.15b | Number of holes knotted | \| \| \| | NKNOT |
| 4.15c | Number of holes patched | \| \| \| | NPATCH |
